# Comparative Efficacy and Safety of Fractional CO_2_ Laser and Gold Microneedling Radiofrequency for Atrophic Acne Scars: A Systematic Review

**DOI:** 10.1111/srt.70345

**Published:** 2026-04-02

**Authors:** Xiaoyan Xiang, Weilong Shuai, Yunzhu Mu

**Affiliations:** ^1^ Department of Dermatology The Affiliated Hospital of North Sichuan Medical College Nanchong Sichuan Province China

**Keywords:** acne scars, fractional CO_2_ laser, fractional microneedle radiofrequency, golden microneedle radiofrequency

## Abstract

**Background:**

Acne is a prevalent dermatological disorder that profoundly affects patients' quality of life, frequently resulting in both physical discomfort and psychological distress. Conventional treatments, including topical agents, oral medications, and chemical peels, are primarily designed to alleviate symptoms but may be insufficient in effectively addressing persistent acne scars. Recent innovations in therapeutic technologies, such as microneedling radiofrequency (RF) and fractional carbon dioxide (CO_2_) laser, have demonstrated significant potential in enhancing skin regeneration and improving the aesthetic appearance of acne scars.

**Materials and Methods:**

Microneedling RF operates by generating mechanical microinjuries in conjunction with RF energy, thereby stimulating dermal remodeling and collagen synthesis. In contrast, fractional CO_2_ laser creates precisely controlled microthermal zones that facilitate wound healing and promote the regeneration of new tissue. This review critically examines the clinical efficacy of these two modalities, exploring their individual mechanisms and comparing treatment outcomes. Additionally, the potential synergistic effects of combining these technologies are discussed.

**Results:**

Both microneedling RF and fractional CO_2_ laser have demonstrated clinical efficacy in treating acne scars. The treatment outcomes, however, may vary depending on patient‐specific factors and treatment parameters. The combined use of these modalities is currently under investigation for its potential to enhance therapeutic effects.

**Conclusion:**

Microneedling RF and fractional CO_2_ laser have proven to be effective in improving acne scars, with promising results in skin regeneration and scar appearance. Future research should focus on refining treatment protocols and exploring the synergistic effects of combining these approaches to optimize clinical outcomes.

## 1 Introduction

Acne is a common chronic inflammatory skin condition that primarily affects the hair follicle‐sebaceous gland unit [1]. It has a considerable global impact, with a reported prevalence of approximately 9.4% of the global population, making it the eighth most prevalent disease worldwide [2]. Adolescence is typically the peak period for acne onset, and hormonal fluctuations, particularly increased androgen levels during puberty, are strongly associated with its development [3]. For example, a survey of 18‐year‐olds revealed acne prevalence rates of 35% in males and 23% in females, highlighting the influence of hormonal differences [4]. Moreover, genetic predisposition plays a significant role in acne development, and regional variations in the prevalence of acne scars are influenced by factors such as genetic differences, skincare practices, and access to effective treatments [5].

Clinically, acne manifests as comedones, pustules, and cysts, often resulting in scarring after the active phase resolves. These scars can range from mild to severe forms, including atrophic, hypertrophic, or keloidal scars [6, 7]. The formation of acne scars is a multifactorial process involving inflammation, tissue damage, and abnormal wound healing responses. As such, treatment options for acne scars remain challenging, and many patients experience unsatisfactory results. Moreover, the psychological burden of acne scars—contributing to low self‐esteem, anxiety, and depression—can significantly impact patient's overall quality of life [8]. Recent advancements in dermatological technologies have led to the development of several treatment modalities for acne scars, with fractional CO_2_ laser therapy and golden microneedle radiofrequency (RF) emerging as two of the most widely used approaches. Fractional CO_2_ lasers utilize targeted laser beams to ablate damaged skin layers, stimulating collagen regeneration and improving scar texture [9]. In contrast, golden microneedle RF combines microneedling with RF energy to induce dermal remodeling and enhance skin repair [10]. Although both treatments have distinct mechanisms of action, efficacy profiles, and potential side effects, they represent promising options for scar management. Personalized treatment plans are essential to achieving optimal outcomes.

This article aims to provide a comparative analysis of the efficacy of fractional CO_2_ laser therapy and golden microneedle RF in treating acne scars. By synthesizing current research, this review seeks to offer valuable insights for clinicians, guiding treatment decisions and improving patient outcomes.

## 2 Methods

### 2.1 Study Design

This study was designed as a systematic review aiming to summarize and compare the clinical efficacy and safety of fractional CO_2_ laser, gold microneedling RF, and their combination in the treatment of atrophic acne scars. The review was conducted and reported in accordance with the Preferred Reporting Items for Systematic Reviews and Meta‐Analyses (PRISMA 2020) guidelines. The PRISMA checklist is provided in Supplementary Table S1, and the study selection process is illustrated in Supplementary S2.

### 2.2 Literature Search Strategy

A comprehensive literature search was conducted in the electronic databases PubMed (MEDLINE), Embase, and Web of Science to identify relevant studies published between January 2014 and December 2024.

The search strategy incorporated both Medical Subject Headings (MeSH) and free‐text terms related to acne scars and energy‐based treatment modalities. The primary search terms included combinations of the following keywords: “acne scars,” “atrophic acne scars,” “fractional CO_2_ laser,” “CO_2_ fractional laser,” “microneedling radiofrequency,” “gold microneedling,” and “fractional radiofrequency.”

The search strategy was adapted for each database as appropriate. In addition, the reference lists of included studies and relevant review articles were manually screened to identify additional eligible publications.

### 2.3 Eligibility Criteria

Studies were included if they met all of the following criteria:

1. Clinical studies, including randomized controlled trials, prospective studies, retrospective studies, and observational studies;
2. Enrollment of patients with atrophic acne scars, including ice‐pick, boxcar, and rolling scar subtypes;
3. Use of fractional CO_2_ laser, gold microneedling RF, or a combination of these modalities as the primary intervention;
4. Reporting at least one extractable clinical outcome related to efficacy or safety.

Studies were excluded if they:

1. Were case reports, conference abstracts, editorials, or narrative reviews;
2. Focused exclusively on non–energy‐based therapies;
3. Included hypertrophic scars or keloids without separate data for atrophic scars;
4. Lacked extractable clinical outcome data.

### 2.4 Study Selection

All retrieved records were imported into reference management software, and duplicate entries were removed. Two reviewers independently screened the titles and abstracts to assess eligibility. Full‐text articles were subsequently reviewed for inclusion based on the predefined criteria. Any discrepancies were resolved through discussion and consensus.

### 2.5 Outcome Measures

Outcome measures were predefined prior to data extraction. The primary outcomes included:

1. Changes in the Echelle d’Évaluation Clinique des Cicatrices d'Acné (ECCA) score, with stratification by scar subtype (ice‐pick, boxcar, and rolling scars);
2. Incidence of post‐inflammatory hyperpigmentation (PIH);
3. Downtime, defined as the number of days required for re‐epithelialization or return to normal daily activities;
4. Patient satisfaction, assessed using validated questionnaires or subjective rating scales.

The secondary outcomes included duration of erythema, pain scores, infection rates, scarring, and other treatment‐related adverse events.

### 2.6 Data Extraction and Synthesis

Extracted information included study design, sample size, patient demographics, acne scar subtype, treatment parameters, clinical efficacy outcomes, and safety profiles. Fitzpatrick skin phototype was extracted when explicitly reported.

Given the substantial heterogeneity in study designs, treatment protocols, and outcome reporting, quantitative meta‐analysis was deemed inappropriate. Therefore, findings were synthesized qualitatively, with emphasis on comparative trends across treatment modalities and scar subtypes.

### 2.7 Risk of Bias Assessment

Formal quantitative risk‐of‐bias assessment was not performed due to the heterogeneity of study designs and outcome measures. However, methodological quality was considered qualitatively during data interpretation, with attention to study design, sample size, outcome assessment methods, and follow‐up duration. Given the predominance of non‐randomized and heterogeneous study designs, formal quantitative tools such as the Cochrane RoB 2 or ROBINS‐I were considered inappropriate.

## 3 Formation Mechanism and Classification of Acne Scars

The formation of acne scars involves a complex interplay of inflammation, follicular damage, and abnormal collagen deposition [11]. During acne development, hair follicles and sebaceous glands are colonized by *Propionibacterium acnes*, which triggers a local immune response and inflammation [12]. *P. acnes* primarily activates the innate immune system through the stimulation of Toll‐like receptors (TLRs), leading to the release of pro‐inflammatory cytokines such as IL‐1, IL‐6, IL‐8, IL‐12, and TNF‐α [13]. Among these, IL‐8 serves as a potent chemotactic factor for neutrophils, recruiting them to the site of infection. In response, neutrophils release lysosomal enzymes that damage the follicular epithelium, causing follicular rupture and exacerbating the inflammatory process [14].

When the inflammation extends into the dermis, it can cause further damage to the hair follicles and surrounding tissues, triggering fibroblast proliferation and activation. This fibroblast activity is typically associated with an increase in collagen synthesis [15]. Depending on the pathological context, collagen may be excessively deposited or insufficiently degraded, both of which contribute to the formation of scars. The extent, duration, and intensity of inflammation, along with its impact on collagen metabolism, determine the type of scar that forms [16]. Chronic inflammation often leads to excessive collagen deposition, resulting in hypertrophic scars or keloids, whereas inadequate inflammation may result in atrophic scars, characterized by significant collagen loss [1, 12]. Therefore, the regulation of inflammation, cytokine levels, and collagen turnover are pivotal factors in the development of acne scars.

**FIGURE 1:**
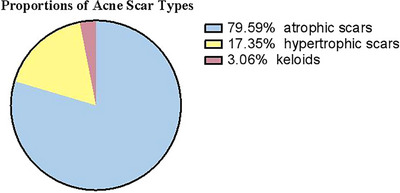
Distribution of atrophic acne scar subtypes. Adapted from previously published literature [17]; figure redrawn by the authors.

**FIGURE 2:**
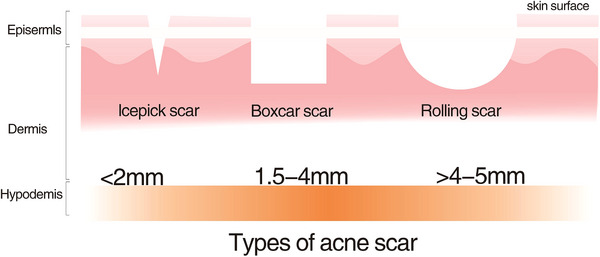
The three types of atrophic acne scars: (A) icepick, (B) boxcar, and (C) roll. Adapted from previously published literature [19]; figure redrawn by the authors.

Histologically, acne scars are commonly classified into three types: atrophic, hypertrophic, and keloidal scars. Atrophic scars, the most prevalent form, constitute the majority of acne scars (The proportions is shown in Figure 1 These scars can be further categorized into ice‐pick scars, rolling scars, and boxcar scars(details summarized on Table 1, Figure 2)) [17, 18, 19].

**TABLE 1:** Classification of atrophic acne scars [19].

Type of acne scar	Morphological description	Typical size and key features
Boxcar scars	Broad, well‐demarcated atrophic scars with sharp vertical edges	Diameter: 1.5–4 mm; well‐defined borders; relatively uniform depth
Ice pick scars	Narrow, deep, V‐shaped scars extending into the deep dermis or subcutaneous tissue	Diameter: <2 mm; marked vertical depth disproportionate to surface width
Rolling scars	Broad, shallow scars with undulating surface contour caused by dermal tethering	Diameter: 4–5 mm; sloping edges; wave‐like appearance

## 4 Acne Scar Treatment

### 4.1 Conventional Therapeutic Approaches for Acne Scarring

Once acne scars have formed, their repair becomes increasingly challenging, emphasizing the importance of early intervention to minimize the risk of scarring. Early acne treatment has been shown to prevent or mitigate the severity of scar formation [19, 20]. By effectively controlling inflammation early on, further acne progression can be halted, which significantly reduces the likelihood of scar development. Common treatment modalities for acne scars include medications, photothermal therapies, tissue fillers, chemical peels, and surgical interventions [21].

### 4.2 Medications

Isotretinoin, a well‐known treatment for acne, reduces sebum production and suppresses inflammation. Although effective in managing active acne, its ability to address deep or severe scars is limited. Common side effects of isotretinoin include dry skin, itching, and peeling [22].

### 4.3 Chemical Peels

Chemical peels work by removing the epidermis to improve skin texture. However, while they can enhance the appearance of superficial scars, they are less effective for deeps cars and may cause side effects such as pigmentation changes, erythema, and heightened skin sensitivity [23].

### 4.4 Tissue Filler Treatments

Substances like hyaluronic acid are effective for treating superficial, atrophic scars. However, these effects are temporary, requiring repeated injections to maintain results. Short‐term side effects include redness, swelling, bruising, and discomfort at the injection site [24].

### 4.5 Photothermal Therapy

In recent years, photothermal therapy, including laser and photodynamic therapy, has emerged as an important treatment for acne scars. CO_2_ laser therapy, in particular, stimulates collagen regeneration through thermal effects, which helps improve skin texture, flatten atrophic scars, and reduce pigmentation [25, 26]. However, CO_2_ fractional laser treatment may cause erythema, edema, scar formation, and pigmentation changes, usually due to thermal damage to the skin's superficial and deeper layers [27].

### 4.6 Microneedling

Microneedling uses tiny needles to create micro‐punctures in the dermis, initiating a healing response that stimulates collagen production. It also promotes the release of growth factors such as TGF‐α, TGF‐β, and Platelet‐Derived Growth Factor [28]. However, microneedling's effectiveness is limited for treating severe or fibrotic scars. Short‐term side effects may include localized redness, pain, and minor infections [29].

### 4.7 RF Therapy

RF therapy generates heat in the deeper layers of the skin and subcutaneous tissues, which promotes keratinocyte and fibroblast proliferation. This leads to increased collagen and hyaluronic acid production and the remodeling of elastin fibers [30]. Although RF therapy is effective for improving acne scars, its limited depth of action may not be sufficient for severe or fibrotic deep scars [31].

### 4.8 Gold Microneedling With RF

Combining microneedling with RF energy stimulates fibroblasts to produce collagen, aiding in the treatment of acne scars. This combined approach has shown promising results in promoting scar healing [32].

## 5 Mechanism of CO_2_ Fractional Laser and Gold Microneedle RF

### 5.1 CO_2_ Fractional Laser Mechanism

In 1983, Anderson introduced the theory of Selective Photothermolysis, which suggests that laser energy can precisely target specific skin chromophores—such as melanin, hemoglobin, or water—using tailored wavelengths and pulse durations. This selective absorption generates localized thermal damage, sparing surrounding healthy tissue, and thereby promoting accelerated tissue repair and regeneration [33].In CO_2_ fractional laser therapy, the laser's 10,600 nm wavelength specifically targets water in the skin tissues, creating microthermal zones (MTZs)—small columns of thermal injury in the skin. These microzones trigger several key mechanisms that contribute to healing and skin regeneration:

1. Activation of the Immune Response: The Localized Thermal Damage Prompts an Immune Response, Attracting Inflammatory Cells to the Treated Area. These Cells Release Cytokines and Stimulate Angiogenesis, Which Accelerates Wound Healing [34]
2. Stimulation of Fibroblasts: The Thermal Injury Within the Dermis Activates Fibroblasts, Enhancing the Synthesis of Collagen and Elastin Fibers. This Process Helps Repair Atrophic Scars and Improves Skin Texture [35]
3. Collagen Remodeling: Heat‐induced Injury Not Only Stimulates New Collagen Production but Also Promotes the Reorganization of Existing Collagen Fibers, Which Enhances Skin Firmness and Elasticity [36]
4. Micro‐Ablation Effect: The Laser's Micro‐ablation Effect Removes Damaged Epidermal Tissue, Facilitating the Regeneration of Healthier Skin Cells [37]. These Combined Mechanisms Make CO_2_ Fractional Laser Therapy a Highly Effective Treatment for Improving Scar Texture and Enhancing Overall Skin Quality

### 5.2 Gold Microneedling RF Mechanism

#### 5.2.1 Dual Mechanism: Synergy of Microneedles and RF Energy

Gold microneedling RF therapy combines microneedling technology with RF energy to deliver controlled thermal energy deep into the dermis [38]. The microneedles create microchannels in the skin, initiating the natural wound healing process by attracting inflammatory cells and growth factors to the treatment area, thus promoting skin regeneration [39, 40]. Concurrently, RF energy induces thermal damage in the dermis, causing immediate collagen contraction and activating fibroblasts. This stimulation leads to the production of new collagen, elastin, and hyaluronic acid [41].

#### 5.2.2 Collagen Regeneration and Tissue Remodeling

The thermal energy delivered by RF not only results in immediate collagen contraction but also triggers long‐term collagen synthesis and remodeling. Over the course of weeks to months, activated fibroblasts produce new collagen and elastin fibers, gradually filling the depressions caused by atrophic scars and improving the skin's overall structure and texture [42, 43]. This ongoing process enhances both skin firmness and elasticity.

#### 5.2.3 Hyaluronic Acid Synthesis and Improved Hydration

In addition to stimulating collagen production, RF energy also boosts the synthesis and retention of hyaluronic acid, a key component for skin hydration. The increased presence of hyaluronic acid improves skin moisture, suppleness, and elasticity, which helps smooth the texture of acne‐scarred skin [31, 41].

## 6 Comparison of the Efficacy of CO_2_ Fractional Laser and Gold Microneedling RF

CO_2_ fractional laser and gold microneedling RF are both widely recognized and effective treatments for acne scars. A study involving 45 patients treated with CO_2_ fractional laser revealed that 60% of participants (27 individuals) experienced good to excellent results 1 month post‐treatment, with minimal side effects and high patient satisfaction [44]. In another study focusing on gold microneedling RF for facial atrophic acne scars, 126 patients showed improvements in symptoms after treatment. Notably, 92 patients (73.0%) reported moderate to significant improvement [45].

Although current research generally supports the effectiveness of both CO_2_ fractional laser and gold microneedling RF in treating acne scars, there are some inconsistencies in the findings and interpretations across studies.

### 6.1 Gold Microneedling RF Shows Superior Treatment Effects and Prognosis Compared to CO_2_ Fractional Laser

Shen Hui et al. divided 64 patients into two groups: the observation group (gold microneedling RF) and the control group (CO_2_ fractional laser). The study found that the observation group had significantly shorter inflammatory exudation time, healing time, scab shedding time, and erythema duration compared to the control group, with statistically significant differences (*p* < 0.05). After treatment, both groups showed significant reductions in the ECCA scores, with the observation group having significantly lower scores than the control group (*p* < 0.05). The total effective rate in the observation group was 93.75%, significantly higher than the control group's 71.88% (*p* < 0.05). The overall incidence of adverse reactions in the observation group was 9.38%, significantly lower than the control groups 43.75% (*p* < 0.05) [46]. Another study also indicated that the experimental group (gold microneedling RF) showed better overall treatment effectiveness, ECCA score improvement, and scab shedding time compared to the reference group (CO_2_ fractional laser), with statistically significant differences (*p* < 0.05) [47]. Wang Luming et al. conducted a controlled analysis on 40 patients with facial atrophic acne scars, comparing the treatment effects of both methods. The results showed that gold microneedling RF had significantly better treatment outcomes compared to CO_2_ fractional laser, with statistically significant differences (*p* < 0.05). Furthermore, gold microneedling RF was also superior in improving patient satisfaction and quality of life [48].

### 6.2 Some Studies Suggest That CO_2_ Fractional Laser May Provide Better Treatment Results Than Gold Microneedling RF

A study involving 177 patients with facial acne scars analyzed the effects of both treatments. The results showed that CO_2_ fractional laser had the best effect in improving acne scars, while gold microneedling RF showed the least effectiveness [49].

### 6.3 Overall Treatment Effects are Similar, but Gold Microneedling RF Shows Advantages Over CO_2_ Fractional Laser

Hendel K treated 15 patients with moderate to severe acne scars using both CO_2_ fractional laser and microneedling RF. After 3 months, both treatments demonstrated a median improvement of 1 point, with the best area achieving a 3‐point improvement (*p* < 0.05). CO_2_ laser treatment resulted in more significant erythema and skin barrier damage (*p* < 0.05), while microneedling RF caused more pain (VAS score:7.0 vs. 5.5), with statistically significant differences (*p* < 0.05) [50].

A total of 50 patients with acne scars were enrolled in Rajput D et al.’s study, which revealed that the CO_2_ group's score improved by 63.41%, from 29.24 to 10.7, and the MNRF group saw a 60.72% improvement, with a reduction from 33.24 to 13.04. The differences between the two groups were statistically significant (*p* < 0.05). In the blinded physician evaluation, four patients in the CO_2_ group showed more than 75% improvement (Grade 4), and 14 showed 51%–75% improvement (Grade 3). In the MNRF group, three patients showed more than 75% improvement (Grade 4), and 12 showed 51%–75% improvement (Grade 3). No significant difference between the groups was observed (*p* > 0.05). Although both treatments showed similar efficacy, CO_2_ fractional laser produced quicker results, while microneedling RF exhibited gradual improvement. Furthermore, microneedling RF had a shorter recovery time, making it more suitable for patients with darker skin tones [51].

A study conducted by Reddy et al. [52] on 30 patients to evaluate the efficacy and safety of both treatments. No statistically significant difference (*p* > 0.05) was found between gold microneedling RF and CO_2_ fractional laser in terms of qualitative and quantitative scar improvement scores. However, patients treated with microneedling RF reported higher satisfaction and no post‐inflammatory pigmentation (*p* < 0.05), a difference that was statistically significant.

Sriram et al. investigated a cohort of 32 patients with skin types III and IV, dividing them into two groups for further treatment. There was no significant difference in final scores after treatment (*p* > 0.05), with both treatments demonstrating statistically similar efficacy. However, in the CO_2_ laser group, two patients (12.5%) developed post‐inflammatory pigmentation (PIH), while no cases of pigmentation were reported in the microneedling RF group [53].

Zhang Lidan et al. administered gold microneedling RF to 42 patients and CO_2_ fractional laser treatment to 47 patients. The overall efficacy rates for the two groups were 92.9% and 89.4%, respectively, with no statistically significant difference (*p* > 0.05). Although the efficacy rates were similar, microneedling RF showed advantages in terms of improvement, patient satisfaction, and wound recovery [54].

Despite CO_2_ fractional laser demonstrating significant efficacy in treating acne scars, Chan N's study found that 55.5% of patients developed post‐inflammatory pigmentation within 1 month after treatment [55]. Consequently, while CO_2_ fractional laser yields faster results, microneedling RF may offer advantages in minimizing side effects and enhancing patient satisfaction.

These clinical differences may largely be attributed to variations in device configuration and treatment parameters. Commonly utilized CO_2_ fractional laser platforms include CO_2_ RE (Syneron Candela), Derma India Futura RF30, and Lutronic ECO_2_, typically operated in ultra‐pulse mode (10,600 nm) with energy settings ranging from 50 to 200 mJ, coverage densities between 5% and 30%, and treatment intervals of approximately 4–8 weeks [46, 51, 52].

In comparison, gold microneedling radiofrequency (GMRF) systems‐such as United (Shenzhen Peninsula Medical Co.), DERMA INDIA MR 16‐2SB, and Infini‐Lutronic‐employ 49 gold‐plated micro‐needles with adjustable penetration depths of 0.5–3.5 mm, power outputs of 6–30 W, and pulse durations ranging from 100–300 ms. RF energy is selectively delivered into the dermis while sparing the epidermis, allowing more controlled dermal remodeling with reduced downtime [43, 44].

For darker skin types, treatment settings should be cautiously adjusted to minimize epidermal thermal injury and the risk of post‐inflammatory hyperpigmentation (PIH), including the use of lower energy levels, increased spacing, and extended treatment intervals. [51] Prior to treatment, before treatment, the skin is cleansed and topical anesthesia (5%–10% lidocaine) is applied for 30–45 min. Cooling systems may be used during CO_2_ laser sessions to reduce discomfort and thermal damage. After treatment, gentle cleansing, regular moisturization, and strict photoprotection are essential to support healing and prevent PIH. Recovery usually occurs within 5–7 days for CO_2_ laser and 24–48 h for MNRF [49]]

Overall, variations in device type, energy settings, and procedural protocols across studies may partly account for the inconsistent efficacy and safety outcomes reported. A concise summary of representative devices and typical treatment parameters is provided in Table 2.

**TABLE 2:** Commonly reported CO_2_ fractional laser and gold microneedling RF devices and treatment parameters.

Treatment modality	Common devices (reported in studies)	Parameter range (light‐medium skin)	Adjustments for darker skin
CO_2_ fractional laser	CO_2_RE(Syneron,Beijing, China);UltraPulse (Lumenis, USA); ECO2‐Lutronic (Korea);Derma India futura RF30 (India)	Energy 30–100 mJ; coverage 5%–30%; spot size 100–300 µm;scan modes Deep/Fusion/Sequential/Random; pulse duration 0.1–10 ms	Energy reduced by 20%–30%, coverage decreased, interval extended 1–2 weeks
Gold microneedling radiofrequency	United(Shenzhen Peninsula,China); Infini‐Lutronic (Korea);Secret RF (Cutera, USA)	Needle length 0.5–3.5 mm; RF power 6–50 W; output time 100–300 ms; 1–3 passes per session	Needle length and power reduced, number of passes decreased to protect epidermis and minimize hyperpigmentation risk

*Note*: Devices and parameters listed represent ranges reported in the literature. Specific treatment settings should be individualized according to patient skin type, scar characteristics, and clinical judgment.

### 6.4 Treatment Effects for Different Types of Acne Scars

A study on 26 patients with facial atrophic acne scars used a half‐face‐controlled experiment. The two sides of the face were randomly assigned to either the microneedling RF or CO_2_ fractional laser group. After treatment, all patients showed significant improvement in acne scars, with significant reductions in the acne scar weight scores (*p* < 0.05). Analysis of different types of scars showed that microneedling RF was significantly better than CO_2_ fractional laser in treating V‐shaped and M‐shaped scars (*p* < 0.05), while there was no significant difference in treating U‐shaped scars (*p* > 0.05). For deeper V‐shaped scars and larger M‐shaped scars, microneedling RF was more effective, whereas for U‐shaped scars, the two treatments showed no significant difference in efficacy [56]. However, this study differs from Li Xiaoyan et al.’s results, which concluded that there was no significant difference in the overall efficacy of the two treatments. Li Xiaoyan et al. found that gold microneedling RF was more effective in treating M‐shaped scars (*p* < 0.05), while CO_2_ fractional laser had an advantage in treating V‐shaped scars (*p* < 0.05) [57]. Furthermore, Majid I's research indicated that CO_2_ fractional laser, as a single treatment method, is particularly effective in improving atrophic acne scars (especially M‐shaped and superficial boxcar scars), but less effective for V‐shaped scars [58]. According to Huang L's study, M‐shaped scars are most responsive to gold microneedling RF treatment, while U‐shaped scars show the greatest improvement during follow‐up [59].As shown in Table 3, the comparison of the two treatments highlights their respective advantages and limitations.

**TABLE 3:** Comparative clinical outcomes of fractional CO_2_ laser and gold microneedling radiofrequency in atrophic acne scars.

Study	N	Overall efficacy (CO_2_ vs. GMRF)	Scar subtype–specific findings	Patient satisfaction	Key adverse events
Hendel K	15	Similar (median improvement +1)	Not reported	Not reported	CO_2_: more erythema and barrier disruption; GMRF: higher pain
Rajput D	50	Comparable (63.4% vs. 60.7%)	Not reported	Not reported	Not reported
Reddy K Y	30	No significant difference	Not reported	Higher with GMRF	No PIH reported in GMRF group
Sriram R	32	No significant difference	Not reported	Not reported	PIH in 12.5% of CO_2_ group
Zhang Lidan	42	Comparable (92.9% vs. 89.4%)	Not reported	Higher with GMRF	Faster wound recovery with GMRF
Hu Yakun	26	No significant difference	GMRF superior for V‐ and M‐shaped scars	Not reported	Not reported
Li Xiaoyan	80	No significant difference	GMRF superior for M‐shaped; CO_2_ superior for V‐shaped scars	Not reported	Not reported

### 6.5 Combination Therapy Outperforms Single Treatments

Multiple studies have shown that the combination of CO_2_ fractional laser and Gold Microneedling RF achieves better results than either treatment alone. Additionally, combination therapy offers significant advantages during the recovery process, with shorter healing times, reduced erythema duration, and less downtime, indicating that it helps alleviate post‐treatment discomfort and accelerates recovery. Furthermore, the combination therapy group experiences fewer adverse reactions, further demonstrating its higher safety profile (summarized on Table 4) [60, 61, 62, 63, 64].

**TABLE 4:** Summary of clinical outcomes of combined CO_2_ fractional laser and microneedling radiofrequency in atrophic acne scars.

Study	Assessment scale	Overall improvement trend	Durability of response	Downtime / tolerability
Li Xiaoyan	ECCA	Marked improvement after combined treatment	Sustained at follow‐up	Transient erythema (∼2 days)
Li Hui	ECCA	Significant reduction in acne scar severity	Maintained during observation period	Mild erythema (∼1 day)
Jiang Yerong	ECCA	Consistent improvement across sessions	Not specifically reported	Short downtime (<2 days)
Tatlıparmak A	ECCA	Substantial clinical improvement	Partial persistence at follow‐up	Erythema; occasional PIH
Mandavia R	ECCA	Moderate‐to‐marked global improvement	Not reported	Temporary erythema and edema

### 6.6 Comparative Efficacy and Safety Considerations

Taken together, existing evidence indicates that combination therapy generally achieves superior clinical improvement compared with single‐modality treatments, particularly in terms of scar texture, depth reduction, and patient‐reported satisfaction. Nevertheless, the reported efficacy of fractional CO_2_ laser and microneedling RF varies across studies. These inconsistencies may be attributed to differences in study design, patient demographics, scar morphology, treatment parameters, and evaluation scales.

Differences in safety profiles have also been observed. Both modalities are generally well tolerated; however, fractional CO_2_ laser has been associated with a higher incidence of post‐inflammatory hyperpigmentation (PIH) and prolonged erythema, especially in individuals with darker skin types (Fitzpatrick IV‐VI). In contrast, microneedling RF delivers energy selectively to the dermis while sparing the epidermis, resulting in shorter downtime and fewer pigmentary alterations. For darker skin types, cautious adjustment of treatment parameters‐such as lower energy levels, increased spacing, and extended treatment intervals‐has been recommended to minimize the risk of epidermal thermal injury and PIH.

These observations are further supported by a recent systematic review and meta‐analysis on striae distensae, a condition with similar dermal remodeling mechanisms. In this study, fractional microneedle radiofrequency (FMR) and fractional CO_2_ laser (FCL) showed comparable efficacy in lesion improvement, as assessed by both clinicians and patients. However, the incidence of PIH was significantly lower in the FMR group compared with the FCL group (OR: 0.24; 95% CI: 0.08–0.70). These findings reinforce the notion that microneedling RF may offer advantages in minimizing pigmentary complications, particularly in darker skin types, consistent with observations in acne scar treatment [65].

Collectively, these findings emphasize that while both modalities are effective, they exhibit distinct safety profiles. Treatment selection should therefore consider patient skin type, scar characteristics, and tolerance for downtime. Standardization of treatment parameters and outcome measures in future studies would further enhance comparability across trials.

### 6.7 Efficacy Analysis of Both Combined With Other Treatment Modalities

#### 6.7.1 Efficacy Analysis of Microneedling RF Combined With Other Treatment Modalities

Poly‐L‐lactic acid (PLLA) microparticles degrade within the skin, triggering a localized inflammatory response that stimulates fibroblasts to produce new collagen, thereby filling the gaps left by scars [66]. In studies by An Min et al. and Hyeong et al., PLLA was combined with microneedling RF to treat acne scars. The results consistently showed that the combined treatment significantly outperformed monotherapy in terms of scar scores, smoothness, size reduction, overall improvement, and patient satisfaction (*p* < 0.05). Additionally, Hyeong performed skin biopsies on several patients, and histological analysis confirmed that PLLA is degradable and promotes the production of collagen and elastin fibers [67, 68].

Platelet‐rich plasma (PRP), which contains growth factors, cytokines, chemokines, and other bioactive molecules, enhances cell proliferation, collagen synthesis, angiogenesis, and soft tissue remodeling, thus aiding in tissue repair and skin regeneration [69]. A controlled study by Kasy Saeed Y M et al., involving 40 patients, demonstrated that the combination of microneedling RF and autologous PRP was highly effective in treating acne scars, with extremely high patient satisfaction [70].

Botulinum toxin, known for its ability to reduce local muscle tension and improve skin elasticity, can also enhance the effectiveness of acne scar treatments. A study by Bai showed that combining microneedling RF with transdermal botulinum toxin delivery is both an effective and safe approach for treating post‐acne scars, particularly in cases of persistent or hypertrophic acne scars. This combined therapy holds promise as a potential treatment option for such cases [71].

#### 6.7.2 CO_2_ Fractional Laser Combined With Other Treatments for Acne Scar Therapy

Gaumond et al. conducted a review on the combination of CO_2_ fractional laser and platelet‐rich plasma (PRP) for acne scar treatment. Their findings indicated that the combination of PRP and laser therapy was significantly more effective than either therapy alone. This combined approach not only enhanced PRP permeability but also promoted tissue repair and skin regeneration through the microchannels created by the laser [72]. However, a study by Priya et al., using a similar study design, found no significant synergistic effect from the combination of intradermal PRP injection and CO_2_ fractional laser in the treatment of acne scars [73].

Recombinant human epidermal growth factor (rhEGF) plays a vital role in promoting the proliferation and differentiation of keratinocytes and fibroblasts, thus improving skin condition [74]. In a controlled study by Peng et al., 15 patients were treated with CO_2_ fractional laser combined with rhEGF. The results showed that the rhEGF group had a significantly better ECCA score (71.11 ± 27.81) compared to the control group (67.78 ± 26.35), with a statistically significant difference (*p* < 0.05) [75]. Furthermore, EGF has been shown to help reduce skin pigmentation following laser treatment [76].

Subcision is a technique in which a fine needle is inserted beneath the skin to cut the fibrous bands that tether the scar to underlying tissue. This procedure releases the scar from deeper tissues, and the hematoma formed stimulates new tissue generation, promoting fibrosis and skin elevation [77]. Li X analyzed the treatment outcomes of 413 patients, comparing the combination therapy (CO_2_ fractional laser combined with subcision) with the control group (CO_2_ fractional laser alone). The combination group had a significantly higher overall efficacy rate of 92.09%, compared to 77.78% in the control group (*p* < 0.05) [78]. Additionally, studies have indicated that CO_2_ laser incision therapy outperforms microneedling RF incision therapy in terms of effectiveness [79].

Research by Kim et al. demonstrated that the combination of CO_2_ fractional laser with isotretinoin, corticosteroids, and adipose‐derived stem cells achieved favorable therapeutic outcomes [80, 81, 82].

## 7 Results

### 7.1 Overview of Included Studies

A total of 21 studies were included in the qualitative synthesis. These studies collectively investigated the clinical efficacy and safety of fractional CO_2_ laser, gold microneedling radiofrequency (MNRF), and combination therapy in patients with atrophic acne scars, encompassing a range of study designs, scar subtypes, treatment protocols, and outcome measures.

The included studies encompassed randomized controlled trials, prospective and retrospective clinical studies, and observational analyses. Study sample sizes varied considerably, reflecting the heterogeneity of clinical practice settings. Atrophic acne scars were commonly classified as ice‐pick, boxcar, and rolling scars, although the depth of subtype‐specific analysis differed across studies. Clinical efficacy was primarily assessed using validated or semi‐quantitative scoring systems, most frequently the Echelle d’Évaluation Clinique des Cicatrices d'Acné (ECCA) score, supplemented by physician global assessments and patient‐reported outcome measures.

### 7.2 Comparative Efficacy of Fractional CO_2_ Laser and Microneedling RF

Across the included studies, both fractional CO_2_ laser and microneedling RF demonstrated clinically relevant improvements in atrophic acne scars. When overall clinical outcomes were compared, most studies did not report a statistically significant difference in total efficacy between the two modalities.

However, several studies suggested differential responses according to scar subtype. Fractional CO_2_ laser tended to achieve greater improvement in deeper and more sharply demarcated scars, particularly ice‐pick or V‐shaped scars, likely reflecting its capacity for deeper tissue ablation and collagen remodeling. In contrast, microneedling RF showed favorable outcomes in boxcar and rolling scars, which may be attributed to its subdermal collagen stimulation with relative preservation of the epidermal layer.

Patient‐reported satisfaction scores were generally comparable between treatment modalities, although some studies reported higher satisfaction with microneedling RF, potentially related to shorter downtime and improved tolerability.

### 7.3 Efficacy of Combination Therapy

Combination therapy involving fractional CO_2_ laser and microneedling RF was evaluated in a subset of the included studies. These studies consistently reported marked reductions in clinical severity scores, with significant improvements from baseline, including reductions in ECCA scores.

Although combination therapy often demonstrated greater absolute improvement compared with baseline, direct head‐to‐head comparisons with monotherapy were limited. Heterogeneity in treatment parameters, session intervals, and outcome assessment tools precluded definitive conclusions regarding superiority over single‐modality approaches. Nevertheless, the available evidence suggests a potential additive or synergistic effect of combination therapy, particularly in patients presenting with mixed atrophic scar subtypes. An evidence‐informed decision matrix summarizing efficacy trends, downtime tolerance, and safety considerations across scar subtypes is presented in Table 5.

**TABLE 5:** Evidence‐informed decision matrix for the treatment of atrophic acne scars using CO_2_ fractional laser and gold microneedling radiofrequency.

Scar subtype	Treatment modality	Expected efficacy	Downtime tolerance	PIH risk consideration^*^	Clinical recommendation
Ice‐pick scars	CO_2_ fractional laser	High (deep collagen ablation)	Moderate–high	Moderate	Preferred
	Microneedling RF	Moderate	Low–moderate	Low	Optional
	Combination	High	High	Moderate	Selected patients
Boxcar scars	CO_2_ fractional laser	Moderate–high	Moderate	Moderate	Preferred
	Microneedling RF	Moderate	Low	Low	Alternative
	Combination	High	Moderate–high	Moderate	Recommended
Rolling scars	CO_2_ fractional laser	Moderate	Moderate	Moderate	Optional
	Microneedling RF	High (subdermal remodeling)	Low	Low	Preferred
	Combination	High	Moderate	Moderate	Recommended

Note: *PIH risk consideration is inferred from reported adverse events and established laser–skin interaction principles, rather than phototype‐stratified comparative trials.

### 7.4 Safety and Adverse Events

Safety outcomes were reported in most of the included studies and are summarized in Table 6.Across all treatment modalities, adverse events were predominantly transient and self‐limiting.

**TABLE 6:** Summary of reported adverse events associated with CO_2_ fractional laser, microneedling, radiofrequency, and combination therapy.

Treatment modality	Common adverse events	Duration of erythema	PIH occurrence	Pain (VAS or reported)	Notes
CO_2_ fractional laser	Erythema, edema, crusting	2–7 days	Reported in some studies (up to ∼12.5%)	Moderate	Higher downtime; careful parameter selection required
Microneedling RF	Erythema, edema	1–3 days	Rarely reported	Moderate–high	Better epidermal preservation
Combination therapy	Erythema, edema; occasional PIH	1–3 days	Occasionally reported	Moderate	No increase in severe adverse events reported

Fractional CO_2_ laser treatment was associated with longer downtime, typically ranging from 2 to 7 days, and a higher reported incidence of post‐inflammatory hyperpigmentation compared with microneedling RF. Commonly reported reactions included erythema, edema, and crusting.

Microneedling RF demonstrated a more favorable safety profile, with shorter downtime generally limited to 1–3 days and post‐inflammatory hyperpigmentation reported infrequently. Pain intensity was described as moderate to high across studies but was comparable between treatment modalities.

Importantly, combination therapy did not appear to increase the incidence of severe adverse events. Reported reactions were similar in type and duration to those observed with monotherapy, although occasional cases of post‐inflammatory hyperpigmentation were described.

Most included studies did not report safety outcomes stratified by Fitzpatrick skin phototype (III‐ VI); therefore, subgroup analysis according to phototype was not feasible. In addition, heterogeneity in study design and adverse event reporting precluded a formal GRADE‐based comparison between combination therapy and single treatment modalities.

### 7.5 Evidence Integration and Clinical Implications

Given the heterogeneity of study designs, patient populations, treatment parameters, and outcome measures, results were synthesized qualitatively. Overall, the integrated evidence indicates that treatment efficacy and tolerability vary according to scar morphology and patient tolerance for downtime.

To facilitate clinical interpretation of these findings, an evidence‐informed decision matrix was developed, integrating scar subtype, expected efficacy, downtime considerations, and safety profiles in Table 5. This matrix provides a structured overview of comparative treatment suitability based on the synthesized evidence.

## 8 Conclusion

CO_2_ fractional laser and gold microneedling radiofrequency (GMRF) each demonstrate distinct therapeutic profiles in the management of atrophic acne scars, supporting their use in different clinical scenarios and patient populations. CO_2_ fractional laser shows consistent efficacy in the treatment of deeper and more severe atrophic scars, often achieving substantial clinical improvement within a relatively short treatment course. However, its use is commonly associated with longer recovery periods and a higher risk of post‐inflammatory hyperpigmentation.

In contrast, GMRF exhibits a more favorable safety profile with shorter downtime, making it particularly suitable for patients with higher safety concerns, increased pigmentation risk, or a preference for minimal disruption to daily activities. Existing evidence suggests that GMRF may be especially effective for more superficial scar subtypes, such as boxcar and rolling scars, by promoting controlled dermal remodeling with reduced treatment‐related morbidity.

Overall, current findings support an individualized approach to acne scar management, in which treatment selection is guided by scar morphology, patient expectations, and tolerance for downtime. Nevertheless, long‐term comparative data remain limited. Future large‐scale, multicenter studies with standardized outcome measures and extended follow‐up are needed to refine treatment algorithms and clarify the durability of clinical outcomes.

Given the heterogeneity of acne scar presentations, combination therapy represents a promising, evidence‐informed strategy that may address a broader spectrum of scar subtypes while balancing efficacy and safety. By integrating complementary treatment modalities and patient‐specific factors, clinicians may achieve more personalized therapeutic outcomes and improved patient satisfaction.

## Funding

This work was supported by grants from the Nanchong City School Cooperation Project (No. 22SXQT0137).

## Ethics Statement

This review article did not involve any studies with human participants or animals conducted by the authors. All data and findings discussed in this manuscript were obtained from previously published literature; therefore, ethical approval and informed consent were not required.

## Conflicts of Interest

Authors Contribution Statement: All authors contribute equal.

## Supporting information




**Supporting Information file 1**: srt70345‐sup‐0001‐SuppMat1.docx

**Supporting Information file 2**: srt70345‐sup‐0002‐SuppMat2.docx

## Data Availability

The data that support the findings of this study are available on request from the corresponding author. The data are not publicly available due to privacy or ethical restrictions.
