# Supplementary material for: Comparative Efficacy and Safety of Fractional CO2 Laser and Gold Microneedling Radiofrequency for Atrophic Acne Scars: A Systematic Review
Source: Skin Res Technol. 2026 Apr 2;32(4):e70345. doi: 10.1111/srt.70345 (PMC13052295; doi:10.1111/srt.70345)
Supplement: Supplementary file 1 — Supporting Information file 1: srt70345‐sup‐0001‐SuppMat1.docx [file SRT-32-e70345-s002.docx]

| **Section and Topic** | **Item #** | **Checklist item** | **Location where item is reported** |
| --- | --- | --- | --- |
| **TITLE** | | |  |
| Title | 1 | Comparative Efficacy and Safety of Fractional CO₂ Laser and Gold Microneedling Radiofrequency for Atrophic Acne Scars: A Systematic Review | p.Ⅰ |
| **ABSTRACT** | | |  |
| Abstract | 2 | This systematic review summarizes and compares the clinical efficacy and safety of CO₂ fractional laser and gold microneedling radiofrequency, as well as their combination, in the treatment of atrophic acne scars. A comprehensive literature search was conducted in major databases between January 2014 and December 2024. Eligible comparative clinical studies were qualitatively synthesized. Outcomes included scar severity improvement, post-inflammatory hyperpigmentation, downtime, and patient satisfaction. The findings suggest that both modalities are effective, with distinct advantages depending on scar subtype, while combination therapy may offer enhanced outcomes in selected patients.” | p.Ⅱ |
| **INTRODUCTION** | | |  |
| Rationale | 3 | Atrophic acne scars remain a challenging sequela of acne vulgaris, with substantial impact on patients’ quality of life. Energy-based devices, including CO₂ fractional laser and microneedling radiofrequency, have been widely used and increasingly compared in clinical practice. However, existing studies report heterogeneous outcomes, parameters, and safety profiles, and a consolidated evaluation of comparative evidence remains limited. A systematic synthesis of available clinical data is therefore warranted to inform treatment selection and optimize individualized management strategies. | p.1 |
| Objectives | 4 | The objective of this systematic review was to qualitatively compare the clinical efficacy and safety of CO₂ fractional laser and gold microneedling radiofrequency in the treatment of atrophic acne scars, and to evaluate the potential benefits of combination therapy based on scar subtype and treatment-related outcomes. | p.1 |
| **METHODS** | | |  |
| Eligibility criteria | 5 | Studies were eligible if they met the following criteria: (1) human clinical studies involving patients with atrophic acne scars; (2) evaluation of CO₂ fractional laser, microneedling radiofrequency, or their combination; (3) comparative study design; and (4) reporting of clinical outcomes such as scar severity scales, post-inflammatory hyperpigmentation, downtime, or patient satisfaction.  Exclusion criteria included animal or in vitro studies, case reports or small case series, studies focusing on active acne rather than scars, reviews or conference abstracts, and studies evaluating non–energy-based treatments only. | p.2 |
| Information sources | 6 | A comprehensive literature search was conducted in PubMed (MEDLINE), Web of Science, Embase, and Scopus between January 2014 and December 2024. Reference lists of relevant articles were also manually screened to identify additional studies. | p.2 |
| Search strategy | 7 | The search strategy combined free-text terms related to acne scars and energy-based treatment modalities, including “acne scars,” “atrophic acne scars,” “CO₂ fractional laser,” and “microneedling radiofrequency.” The detailed search strategy for PubMed is provided in the Supplementary Materials. | p.2 |
| Selection process | 8 | All retrieved records were imported into reference management software, and duplicate records were removed. Two reviewers independently screened titles and abstracts for eligibility. Full-text articles were subsequently assessed to determine final inclusion. Disagreements were resolved through discussion. | p.2,3 |
| Data collection process | 9 | Data were independently extracted by two reviewers using a predefined data extraction form. When outcome data were unclear or incomplete, the information was interpreted based on the reported results without contacting original authors. | p.3 |
| Data items | 10a | The primary outcomes included changes in acne scar severity assessed by validated scales (e.g., ECCA, Goodman and Baron scale), incidence of post-inflammatory hyperpigmentation, duration of downtime, and patient-reported satisfaction. | p.2 |
|  | 10b | Additional data items included study design, sample size, scar subtype, treatment parameters, number of treatment sessions, follow-up duration, and reported adverse events | p.3 |
| Study risk of bias assessment | 11 | A formal risk of bias assessment was not performed due to heterogeneity in study designs, outcome measures, and reporting formats among the included studies. | p.3 |
| Effect measures | 12 | Effect measures were reported descriptively. Quantitative pooling of effect estimates was not performed.pecify for each outcome the effect measure(s) (e.g. risk ratio, mean difference) used in the synthesis or presentation of results. | Not applicable (qualitative synthesis only) |
| Synthesis methods | 13a | Studies evaluating CO_2_ laser or microneedling radiofrequency in combination with additional treatment modalities (e.g., PRP, subcision) were discussed narratively and were not included in the primary qualitative synthesis. | p.1 |
|  | 13b | When summary statistics were incompletely reported, data were extracted as presented without statistical transformation. No imputation or data conversion was performed. | Not applicable |
|  | 13c | Results of individual studies were tabulated using structured summary tables. Key outcomes, including scar severity improvement and adverse events, were described narratively. | Table2 and table 3;p.6-p.10 |
|  | 13d | A qualitative synthesis approach was chosen due to substantial heterogeneity in study design, outcome measures, and treatment protocols. Meta-analysis was not performed as pooling of results was deemed inappropriate. | Not applicable |
|  | 13e | No formal methods were used to explore heterogeneity among study results. | Not applicable |
|  | 13f | Sensitivity analyses were not conducted. | Not applicable |
| Reporting bias assessment | 14 | Reporting bias was not formally assessed. | Not applicable |
| Certainty assessment | 15 | The certainty of evidence was assessed qualitatively based on study design, consistency of findings, and reported outcomes. | Not applicable |
| **RESULTS** | | |  |
| Study selection | 16a | A total of 320 records were identified through database and manual searches. After removal of duplicates, 238 records were screened by title and abstract, and 52 full-text articles were assessed for eligibility. Ultimately, 21studies were included in the qualitative synthesis. The study selection process is summarized in the PRISMA flow diagram. | PRISMA flow diagram |
|  | 16b | Several studies were excluded after full-text review because they lacked comparative data, did not specifically evaluate atrophic acne scars, or focused on non–energy-based treatments only. Examples of excluded studies and reasons for exclusion are provided in the Supplementary Materials. | Not applicable |
| Study characteristics | 17 | The characteristics of the included studies, including sample size, intervention type, outcome measures, and main findings, are summarized in the corresponding tables | Not applicable |
| Risk of bias in studies | 18 | Risk of bias was not formally assessed. | Not assessed |
| Results of individual studies | 19 | Individual study results regarding efficacy, safety, and patient-reported outcomes are presented descriptively in structured tables. | Not applicable |
| Results of syntheses | 20a | The included studies were mainly prospective or retrospective comparative clinical studies involving patients with atrophic acne scars. Treatment modalities included CO₂ fractional laser, microneedling radiofrequency, and combination therapy. Outcome measures varied across studies, including ECCA score, Goodman and Baron grading system, investigator global assessment, and patient satisfaction scales.  A formal risk of bias assessment was not conducted due to heterogeneity in study designs and outcome reporting; therefore, risk of bias was assessed qualitatively based on study methodology and reporting transparency. | p.11,p.12 |
|  | 20b | No statistical synthesis or meta-analysis was performed due to substantial methodological and clinical heterogeneity among the included studies. Therefore, summary estimates, confidence intervals, and measures of statistical heterogeneity were not applicable. | Not applicable |
|  | 20c | No formal investigations of heterogeneity, such as subgroup analysis or meta-regression, were conducted. | Not applicable |
|  | 20d | Sensitivity analyses were not performed. | Not applicable |
| Reporting biases | 21 | Reporting bias was not assessed. | Not assessed |
| Certainty of evidence | 22 | Overall certainty of evidence was considered moderate based on qualitative assessment. | Not applicable |
| **DISCUSSION** | | |  |
| Discussion | 23a | This systematic review suggests that both CO₂ fractional laser and microneedling radiofrequency are effective treatment options for atrophic acne scars. CO₂ fractional laser appears to provide more pronounced improvement in deep and severe scars, whereas microneedling radiofrequency is associated with shorter downtime and a more favorable safety profile. Combination therapy demonstrated consistent improvement across different scar subtypes, supporting its potential role in selected patients. | p.13 |
|  | 23b | Several limitations of the included evidence should be acknowledged. Most studies had relatively small sample sizes and short follow-up durations. Outcome measures and scar classification systems were inconsistent across studies, limiting direct comparison. In addition, few studies stratified results according to specific atrophic scar subtypes. | p.13 |
|  | 23c | This review has limitations inherent to its methodology. A formal risk of bias assessment and quantitative meta-analysis were not performed due to heterogeneity in study designs and reported outcomes. Additionally, only studies published in peer-reviewed journals were included, which may introduce publication bias. | Not applicable |
|  | 23d | The findings of this review support individualized treatment selection based on scar subtype, patient tolerance, and downtime considerations. Future research should focus on well-designed randomized controlled trials with standardized outcome measures and longer follow-up to better define the comparative effectiveness of these modalities and optimize combination treatment strategies. | p.13 |
| **OTHER INFORMATION** | | |  |
| Registration and protocol | 24a | This systematic review was not registered, and a formal protocol was not prepared. | Not applicable |
|  | 24b | A formal review protocol was not prepared | Not applicable |
|  | 24c | Not applicable. | Not applicable |
| Support | 25 | This work was supported by grants from the Nanchong City School Cooperation Project (No. 22SXQT0137) | p.14 |
| Competing interests | 26 | The authors declare no competing interests. | p.14 |
| Availability of data, code and other materials | 27 | All data generated or analyzed during this study are included in this published article and its supplementary materials. | Not applicable |

*From:*  Page MJ, McKenzie JE, Bossuyt PM, Boutron I, Hoffmann TC, Mulrow CD, et al. The PRISMA 2020 statement: an updated guideline for reporting systematic reviews. BMJ 2021;372:n71. doi: 10.1136/bmj.n71. This work is licensed under CC BY 4.0. To view a copy of this license, visit <https://creativecommons.org/licenses/by/4.0/>
