# Supplementary material for: Comparative Efficacy and Safety of Fractional CO2 Laser and Gold Microneedling Radiofrequency for Atrophic Acne Scars: A Systematic Review
Source: Skin Res Technol. 2026 Apr 2;32(4):e70345. doi: 10.1111/srt.70345 (PMC13052295; doi:10.1111/srt.70345)
Supplement: Supplementary file 2 — Supporting Information file 2: srt70345‐sup‐0002‐SuppMat2.docx [file SRT-32-e70345-s001.docx]

**Identification of studies via databases and registers**

Records removed *before screening*:

Duplicate records removed (n =85 )

Records marked as ineligible by automation tools (n =0 )

Records removed for other reasons (n =0 )

Records identified from*:

Databases (n =412 )

Registers (n =0 )

**Identification**

Records screened

(n = 327)

Records excluded**

(n = 253)

Reports sought for retrieval

(n =74)

Reports not retrieved

(n = 0)

**Screening**

Reports assessed for eligibility

(n = 74)

Reports excluded:

Reason 1 No extractable clinical outcome data (n = 20)

Reason 2 Non–energy-based interventions (n = 13)

Reason 3 Mixed scar types without stratified data (n = 10)

etc.

Reason 4 Duplicate or overlapping populations (n = 10)

Studies included in review

(n = 21)

Reports of included studies

(n = 0)

**Included**

*Consider, if feasible to do so, reporting the number of records identified from each database or register searched (rather than the total number across all databases/registers).

**If automation tools were used, indicate how many records were excluded by a human and how many were excluded by automation tools.

Source: Page MJ, et al. BMJ 2021;372:n71. doi: 10.1136/bmj.n71.

This work is licensed under CC BY 4.0. To view a copy of this license, visit <https://creativecommons.org/licenses/by/4.0/>
